# Virtual reality-based interventions for the rehabilitation of vestibular and balance impairments post-concussion: a scoping review

**DOI:** 10.1186/s12984-023-01145-4

**Published:** 2023-03-03

**Authors:** Soraya J. LeMarshall, Lachlan M. Stevens, Nicholas P. Ragg, Leia Barnes, Jacinta Foster, Elisa F. D. Canetti

**Affiliations:** 1 Doctor of Physiotherapy, Faculty of Health Sciences and Medicine, Bond University, Gold Coast, Australia; 2 Integrated Specialist ENT Service, Logan Hospital, Meadowbrook, Australia; 3 Tactical Research Unit, Bond University, Gold Coast, Australia

**Keywords:** Virtual reality, Vestibular, Balance, Concussion

## Abstract

**Background:**

Concussions and mild traumatic brain injuries are the most common causes of physical and cognitive disability worldwide. Concussion can result in post-injury vestibular and balance impairments that can present up to five years post initial concussion event, ultimately affecting many daily and functional activities. While current clinical treatment aims to reduce symptoms, the developing use of technology in everyday life has seen the emergence of virtual reality. Current literature has failed to identify substantial evidence regarding the use of virtual reality in rehabilitation. The primary aim of this scoping review is to identify, synthesise, and assess the quality of studies reporting on the effectiveness of virtual reality for the rehabilitation of vestibular and balance impairments post-concussion. Additionally, this review aims to summarise the volume of scientific literature and identify the knowledge gaps in current research pertaining to this topic.

**Methods:**

A scoping review of six databases (PubMed, Embase, CINAHL, ProQuest, SportDiscus, Scopus) and a grey literature (Google Scholar) was conducted using three key concepts (virtual reality, vestibular symptoms, and post-concussion). Data was charted from studies and outcomes were categorised into one of three categories: (1) balance; (2) gait; or (3) functional outcome measures. Critical appraisal of each study was conducted using the Joanna Briggs Institute checklists. A critical appraisal of each outcome measure was also completed utilising a modified GRADE appraisal tool to summarise the quality of evidence. Effectiveness was assessed using calculations of change in performance and change per exposure time.

**Results:**

Three randomised controlled trials, three quasi-experimental studies, three case studies, and one retrospective cohort study were ultimately included, using a thorough eligibility criteria. All studies were inclusive of different virtual reality interventions. The ten studies had a 10-year range and identified 19 different outcome measures.

**Conclusion:**

The findings from this review suggests that virtual reality is an effective tool for the rehabilitation of vestibular and balance impairments post-concussion. Current literature shows sufficient but low level of evidence, and more research is necessary to develop a quantitative standard and to better understand appropriate dosage of virtual reality intervention.

**Supplementary Information:**

The online version contains supplementary material available at 10.1186/s12984-023-01145-4.

## Background

Concussions and mild traumatic brain injuries (mTBI) are the most prevalent cause of physical and cognitive disability worldwide, affecting approximately 450–600 in every 100,000 people each year [1]. Although the terms concussion and mTBI have traditionally been used interchangeably by most medical practitioners and within the scientific literature, there has been a recent emphasis in some medical specialties towards classifying concussion as a less severe form of mTBI [2]. However, currently there are no distinct symptom profiles, diagnostic criteria, or objective biomarkers that distinguish concussion from mTBI [2]. Therefore, for the purposes of this study, the two terms will be used interchangeably. Concussions can arise from various biomechanical means and events including a blow to the head, face, or neck, and may result in an immediate and transient loss of consciousness, accompanied by periods of amnesia [3]. Studies demonstrate 5% to 58% of individuals who sustain a concussion suffer from persistent symptoms and limitations that affect many daily and functional activities [4]. These symptoms vary widely, commonly including headaches, visual disturbances, decreased concentration, fatigue and vestibular impairment [1, 5, 6].

Concussion can result in a high degree of post-injury vestibular impairment, with dizziness reported in ~ 39–90% of concussion cases [7–10], and still present in up to 25–32.5% of cases after 1–5 years of the initial concussion event. [7, 9] It is no surprise that imbalance is also frequently reported post-concussion, with correlations to impaired gait and other functional activities [11]. Vestibular and balance impairment post-concussion significantly increase return to school and return to sport times compared to those with concussion and no vestibular symptoms [12], with subjective complaints lasting from days to years [4]. Current clinical rehabilitation and management of symptoms employed by health professionals aim to reduce such symptoms and ultimately optimise return to work, sport and activities of daily living. Traditional vestibular rehabilitation such as gaze stabilization exercises standing and dynamic balance exercises, and canalith repositioning manoeuvres, are utilised depending on the patient’s presenting symptoms post-concussion [7]. Given the developing use of technology in everyday life, recently there has been a noticeable increase in the use of technology, specifically virtual reality (VR), for the rehabilitation of vestibular impairments, in clinical rehabilitation settings [13–18].

VR is an emerging form of rehabilitation and has been shown to be effective in treating a variety of vestibular and balance impairments [19]. The control and customisation over the VR technology system allows for gradual exposure to environmental stimuli in a safe, controlled, and reproducible environment [20]. Current studies available in the literature suggest that VR rehabilitation has been shown to be more effective than traditional forms of therapy for patients with vestibular and balance impairments [20, 21]. Patients using the technology report increased enjoyment and motivation, less fatigue, better adherence, and an increased ability to tolerate greater cognitive load within VR environments [19–23]. Furthermore, VR technology can provide greater individualised rehabilitation training programs when compared with traditional rehabilitation training. [24, 25]

Prior research [26–30] has established VR as a common assessment tool for identifying vestibular and balance impairments post-concussion, however to date, there has been no research collating or synthesising the use and effectiveness of VR as a rehabilitation modality for these impairments. Moreover, current literature has failed to identify substantial evidence regarding the use of VR in rehabilitating patients’ post-concussion for vestibular and balance impairments [31, 32]. Therefore, the primary aim of this scoping review is to identify, synthesise, and assess the quality of studies reporting on the effectiveness of VR for the rehabilitation of vestibular and balance impairments post-concussion. Secondly, this review aims to summarise the volume of scientific literature and identify the knowledge gaps in current research pertaining to this topic.

## Methods

### Protocol and registration

Prior to commencement of this review, a protocol was developed outlining methodology and eligibility criteria in accordance to Preferred Reporting Items for Systematic Reviews and Meta-Analyses (PRISMA) [33] protocols (PRISMA-P) [34]. The protocol was registered with the Open Science Framework (OSF), registration: 0.17605/OSF.IO/GESBN, and was guided by the PRISMA for Scoping Reviews (PRISMA-ScR) [35].

### Eligibility criteria

To ensure suitability of selected studies, an inclusion and exclusion criteria was established and adhered to throughout the screening process. Studies were included if they met the following criteria: (a) virtual reality used as a treatment; (b) published, peer-reviewed, full-text research studies in English, Spanish, Portuguese and French, with the full text available; (c) studies reporting on treatment and management of vestibular symptoms; (d) human participants (male or female) of any age, from any background; (e) patients diagnosed with any form of concussion or mild traumatic brain injury. Studies were excluded if they met the following criteria: (a) virtual reality was used as an assessment; (b) patients presented with other co-morbidities affecting vestibular and balance function; (c) systematic reviews, conference abstracts, non-peer reviewed studies and dissertations; (d) animal studies; (e) studies reporting on only cognitive and/or executive function.

### Information sources and search strategy

A comprehensive search across six databases (PubMed, Embase, CINAHL, ProQuest, SPORTDiscus, Scopus) was conducted using the identified keywords and index terms for each database information source. The search terms were developed in conjunction with the Bond University librarian and refined using SearchRefinery Tool on Systematic Review Accelerator (SRA) [36] to ensure their suitability. Key search terms were developed based on the concepts of virtual reality, vestibular symptoms, and post-concussion. Due to the inconsistent use of terms in the literature and to maximise the chances of capturing all relevant articles in this scoping review, participants diagnosed with either mTBI or concussion were included. An example of the search terms for PubMed database is provided in Table 1. The search terms were modified to suit each database using Polyglot Search [37] on SRA and relevant MESH terms were manually added for each database (Additional file 1). No filters or limitations were applied to the search. In addition to the six databases, a grey literature search was conducted through Google Scholar, screening for peer reviewed published articles only, where the first 100 records were exported and screened. This was done to ensure all literature was considered and captured. Search was finalised on the 10^th^ of November 2021.

**Table 1:** Search terms used on PubMed on the 10th of November 2021

Database	Search terms
PubMed	("virtual reality"[tiab] OR "immersive technology"[tiab] OR "augmented reality"[tiab] OR "computer simulation"[tiab] OR simulation[tiab] OR "Virtual Reality"[Mesh]) AND (concussion[tiab] OR "traumatic brain injury"[tiab] OR (head[tiab] AND injury[tiab] OR trauma[tiab]) OR "Brain Concussion"[Mesh] OR post-concussion[tiab] OR TBI[tiab]) AND (vestibular[tiab] AND Disorders[tiab] OR therap*[tiab] OR rehabilita*[tiab] OR management[tiab])

### Study selection

All search results were imported to a reference management software (EndNote 20, Clarivate Analytics), and duplicates were removed in SRA DeDuplicator [36]. Initially, three authors (SL, NR, LS) concurrently screened the first 100 records by title and abstract. Discrepancies were resolved through discussion with the senior author (EC), ensuring a stringent screening process. The remaining records were split between the three authors (SL, NR, LS) and screened by title and abstract independently, using the agreed inclusion and exclusion criteria. Records were then collated and categorised on EndNote, into ‘require full text’ or ‘excluded’. Full texts were obtained for these records and screened by two authors (NR, LS) independently to minimise selection bias, with the third author (SL) resolving any disputes where a unanimous decision could not be reached. Excluded records were sorted into folders titled with their corresponding reasons for exclusion. Studies selected for inclusion were agreed upon after discussion between all three authors (SL, NR, LS) and readied for data charting.

### Data charting and data items

Once the included studies were selected and appraised, data charting was undertaken by three authors (SL, NR, LS), independently. Data was charted and exported onto an excel spreadsheet. The data charted from the studies included the following data items: author; year; study characteristics (study design, level of evidence based on the Oxford Centre for Evidence-Based Medicine 2011 scale [38], population/sample size, context of study, type of virtual reality, intervention, outcome of interest); outcome measures; and the key findings.

As types of VR are varied, the authors classified VR into three classes according to the level of immersion: (1) immersive, (2) semi-immersive, and (3) non-immersive VR. Immersive VR was defined as a fully immersive experience encasing both audio and visual perception and eliminating all outside information to the user [39]. Semi-immersive VR consisted of embodying objects into a scene of reality, using three-dimensional environments while users remain connected to the real-world surroundings [39]. Non-immersive VR consisted of a standard monitor or screen placed in front of the user, displaying the virtual environment. [39]

### Critical appraisal of individual sources of evidence

To assess and analyse the quality of scientific literature, the included studies were critically appraised using the Joanna Briggs Institute (JBI) checklists [40] for randomised control trials (RCT), cohort studies (CHS), quasi-experimental studies (QES), and case studies (CS). To minimise any bias, the JBI critical appraisal was completed by two authors (SL, LS) independently. The Critical Appraisal Scores (CAS) were finalised, and qualitative ratings were proposed based on the criteria developed by Kennelly and collegues [41]. To allow comparison between the different checklists, a percentage score was calculated by dividing the sum of total points by the total possible points in each checklist. The CAS criteria categorised  < 45.4% signifying “poor” methodological quality, 45.4% to 61% demonstrating “fair” methodological quality, and > 61% showing “good” methodological quality. A Cohen’s Kappa coefficient was calculated using Statistical Package for Social Sciences (SPSS Version 27, IBM) to provide a level of interrater agreement. Authors interpreted scores based on the following criteria: Kappa scores of 0.0–0.2 slight agreement, 0.21–0.40 fair agreement, 0.41–0.60 moderate agreement, 0.61–0.80 substantial agreement and 0.81–1.00 almost perfect agreement. [42]

### Synthesis of results

To overcome heterogeneity in reported outcome measures, and to best represent the data in the selected studies, outcomes were categorised into one of three categories: (1) balance; (2) gait; or (3) functional outcome measures. For each outcome measure, change in performance was reported as either ‘improved’; ‘declined’; or ‘no change’, by assessing the difference in pre versus post intervention. An ‘improved’ performance was classified when the post intervention score had improved compared to the pre intervention score. Average change per hour of exposure was considered by exploring the change in performance per hour of active intervention. This value was further calculated to a percent (%). Furthermore, the authors calculated a simple percent (%) average change of each outcome measure from pre to post results. A modified Grading of Recommendations, Assessment, Development and Evaluation (GRADE) system was implemented and performed by one author (LS) to quantify quality of evidence across critical outcome measures. Articles were categorised as high (RCT’s), moderate (QES), low (CHS) or very low quality (CS) regarding to their study type. Each article was then appraised focusing on factors listed in table 5.2 and 5.3 in the GRADE handbook [43], and overall article quality was adjusted accordingly. Quality of evidence for each outcome measure was then calculated by averaging quality of evidence scores across all articles that included that specific outcome measure, rounding up to the nearest categorical level (high, moderate, low, very low). This aimed to simplify the findings of this scoping review, providing guidance to inform practice and future research.

### Data availability

The authors confirm that the data supporting the findings of this study are available within the article and its Additional file 1.

## Results

### Selection of sources of evidence

**Fig. 1:**
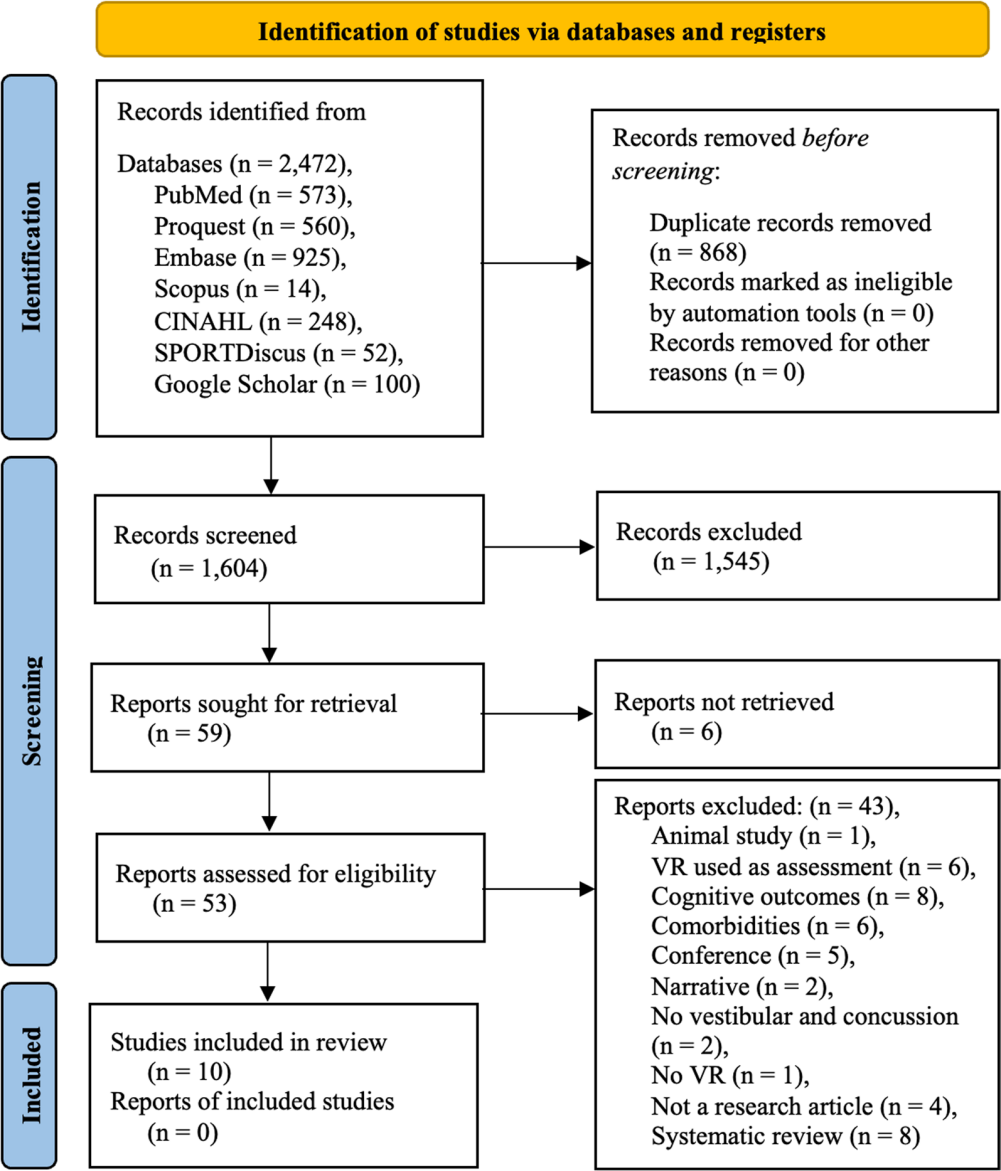
PRISMA [33] Flow diagram

The search identified 2472 records from the selected databases. After removal of duplicates, 1604 records remained for screening. Title and abstract screening excluded 1545 records. Of those remaining, 59 reports remained to be assessed in full text, six of which were unable to be retrieved after attempts to retrieval from both the senior author (EC) and the university’s librarian. In full text screening, 43 reports were excluded with reasons recorded, and ten studies [4, 20, 44–51] were included in this review (Fig. 1).

### Characteristics of sources of evidence

All studies utilised VR-based interventions and were published within a 10-year range. Of the ten studies, one [20] used an immersive VR, seven [4, 44, 46–50] used semi-immersive VR, and two [45, 51] used non-immersive VR as an intervention. All seven studies employing semi-immersive VR intervention utilised the Computer Assisted Rehabilitation Environment (CAREN) technology. The context of which all interventions of the included studies were undertaken was either a medical centre [4, 50], rehabilitation centre [20, 44, 45] or clinical setting. [46–49, 51]

The types of study designs which made up the ten included sources of evidence consisted of RCT [4, 45, 50], retrospective cohort studies [44], quasi-experimental studies, [20, 48, 51] and case studies [46, 47, 49]. Using the Oxford rating scale, [38] six studies [4, 30, 44, 45, 48, 50] were graded as level 2 evidence, one study [44] graded as level 3 evidence, and three studies [46, 47, 49] graded as level 4 evidence quality.

Critical appraisal and data charting was performed on all ten [4, 20, 44–51] studies. The characteristics of these included studies are presented in Table 2.

**Table 2:** Study characteristics of the ten critically appraised studies

Author and year	Study design	Level of evidence (Oxford) [38]	Sample size	Sample size characteristics	Context	VR definition	Outcome of interest
Cano Porras et al. 2019 [44]	Retrospective Cohort Study	Level 3	n = 167	Males = 99 Females = 63 Mean Age = 63.8	Centre of Advanced Technologies in Rehabilitation (CATR) at the Sheba Medical Centre	Semi-Immersive	10 Metre Walk Test Timed Up and Go Four Square Step Test Berg Balance Scale Mini Balance Evaluation Test
Cuthbert et al. 2014 [45]	Randomized Controlled Trial	Level 2	n = 20	Males = 13 Females = 7 Median Age = 31.0	Inpatient Rehabilitation Facility	Non-Immersive	Berg Balance Scale Functional Gait Assessment
De Luca et al. 2019 [46]	Case Report	Level 4	n = 1	Male Age = 15	Supervised Clinical Setting	Semi-Immersive	Tinetti’s Mobility Test Tinetti's Balance Test Functional Independence Measure—Motor Abilities
Gottshall & Sessoms, 2015 [47]	Case Report	Level 4	n = 1	Male Age = 41	Naval Health Research Center (NHRC)	Semi-Immersive	Dizziness Handicap Inventory Sensory Organisation Test Activities-Specific Balance Confidence Scale Functional Gait Assessment
Gottshall, Sessoms and Bartlett 2012 [48]	Quasi-Experimental Design	Level 2	n = 4	Not Specified	Naval Health Research Center (NHRC) Warfighter Performance Laboratory	Semi-Immersive	Sensory Organisation Test Functional Gait Assessment Dizziness Handicap Inventory Activities-Specific Balance Confidence Scale
Lubetzky et al. 2020 [20]	Quasi-Experimental Design	Level 2	n = 15	Males = 10 Females = 5 Mean Age = 38.0	Vestibular Rehabilitation Clinic	Immersive	Visual Vertigo Analogue Scale Dizziness Handicap Inventory Activities-Specific Balance Confidence Scale Eight Foot Up and Go Four-Square Step Test
Rábago and Wilken 2011 [49]	Case Report	Level 4	n = 1	Male Age = 31	Military Performance Laboratory	Semi-Immersive	Single Leg Stance Test Step Variability
Sessoms et al. 2015 [50]	Randomised Control Trial	Level 2	n = 24	Gender Not Specified Mean Age = 29.7	Naval Medical Center San Diego (NMCSD)	Semi-Immersive	CAREN Specific Outcome Measures - Self Selected Walking Speed - Boat Steering Task Score
Sessoms et al. 2021 [4]	Randomised Control Trial	Level 2	n = 25	Gender Not Specified Mean Age = 30.4	Military Medical Centre	Semi-Immersive	Activities-Specific Balance Confidence Scale Dizziness Handicap Inventory Sensory Organisation Test Functional Gait Assessment
Ustinova et al. 2014 [51]	Quasi-Experimental Design	Level 2	n = 15	Males = 10 Females = 5 Mean Age = 30.6	Supervised Clinical Setting	Non-Immersive	Berg Balance Scale Functional Gait Assessment Test Functional Reach Test

### Critical appraisal within sources of evidence

A Cohen’s Kappa analysis revealed a coefficient value of 0.81, indicating ‘almost perfect’ agreement between reviewers [42]. The average CAS for RCT’s [4, 45, 50] were 65%, and 55% for the cohort study’s [44], signifying fair methodological quality for both study designs. The average appraisal score for the quasi-experimental study [51] was 72% and the case reports’ average CAS score was 88%, signifying good methodological quality for both study designs.

Common deficits regarding the methodological quality amongst the RCT’s [4, 45, 50] appraised were a failure to conceal group allocation or blind assessors to treatment. In the cohort study [44] appraisals and follow-ups were commonly not completed or justified and no control group was included. The case reports [46, 47, 49] did not identify or describe adverse events, and none of the quasi-experimental studies [12, 48, 51] included a control group.

### Synthesis of results

Results from individual sources of evidence are displayed in Additional file 2. From the ten included studies, [4, 20, 44–51] nineteen different outcome measures were extracted. Nine outcomes were categorised under balance, four were categorised under gait, and six were categorised under functional (Table 3). Outcome measures common amongst multiple studies include Berg Balance Scale (BBS), Functional Gait Assessment (FGA), Activities-Specific Balance Confidence Scale (ABC Scale), Dizziness Handicap Inventory (DHI), Sensory Organisation Test (SOT) and Four-Square Step Test (FSST). BBS was used in studies of all three types of VR; FGA was seen in studies of immersive and non-immersive VR; while the ABC Scale, DHI, SOT and FSST were used in immersive and semi-immersive VR.

**Table 3:** Outcome measures reported in the  ten included studies

	Cano Porras et al. 2019 [44]	Cuthbert et al. 2012 [45]	De Luca et al. 2019 [46]	Gottshall and Sessoms 2015 [47]	Gottshall et al. 2012 [48]	Lubetzky et al. 2020 [20]	Rábago and Wilken 2011 [49]	Sessoms et al. 2015 [50]	Sessoms et al. 2021 [4]	Ustinova et al. 2014 [51]
**Balance**										
Activities-Specific Balance Confidence Scale	–	–	–	✓	✓	✓	–	–	✓	–
Dizziness Handicap Inventory	–	–	–	✓	✓	✓	–	–	✓	–
Sensory Organisation Test	–	–	–	✓	✓	–	–	–	✓	–
Four Square Step Test	✓	–	–	–	–	✓	–	–	–	–
Berg Balance Scale	✓	✓	–	–	–	–	–	–	–	✓
Mini Balance Evaluation Test	✓	–	–	–	–	–	–	–	–	–
CAREN Specific—Boat Steering Task Score	–	–	–	–	–	–	–	✓	–	–
Tinetti’s Balance Test	–	–	✓	–	–	–	–	–	–	–
Single Leg Stance Test	–	–	–	–	–	–	✓	–	–	–
**Gait**										
Functional Gait Assessment	–	✓	–	✓	✓	–	–	–	✓	✓
10 Metre Walk Test	✓	–	–	–	–	–	–	–	–	–
CAREN Specific—Self Selected Walking Speed	–	–	–	–	–	–	–	✓	–	–
Step Variability	–	–	–	–	–	–	✓	–	–	–
**Functional**										
Timed Up and Go	✓	–	–	–	–	–	–	–	–	–
Functional Reach Test	–	–	–	–	–	–	–	–	–	✓
Tinetti’s Mobility Test	–	–	✓	–	–	–	–	–	–	–
Functional Independence Measure—Motor Abilities	–	–	✓	–	–	–	–	–	–	–
Visual Vertigo Analogue Scale	–	–	–	–	–	✓	–	–	–	–
Eight Foot Up and Go	–	–	–	–	–	✓	–	–	–	–

All outcome measures demonstrated an overall positive effect. Improvements were seen across all 19 outcome measures from pre to post intervention and in percent change per hour of exposure. Modified GRADE scores for each outcome measure ranged from very low to moderate quality, recorded in Table 4.[43] The outcome measures that achieved a moderate grading were the ABC Scale, DHI, BBS and FGA, in which three of these four outcome measures were categorised as balance. Only one moderate grading was seen in gait (FGA), with the rest being of low or very low quality. Functional outcome measures all yielded low or very low quality of evidence. The two outcome measures that received the greatest improvements from pre to post intervention (tinetti balance test; 100% improvement, single leg stance test; 214% improvement), were both graded with very low quality of evidence.

**Table 4:** Synthesis of results

Outcome measure	# Studies (Total # of Participants)	Overall effect (Average % Change)	Quality of evidence (GRADE)
**Balance**			
Activities-Specific Balance Confidence Scale (ABC Scale)	4 (45)	+ (48%)	Moderate
Dizziness Handicap Inventory (DHI)	4 (45)	+ (70%)	Moderate
Sensory Organisation Test (SOT)	3 (30)	+ (12%)	Low
Four Square Step Test (4SST)	2 (17)	+ (52%)	Low
Berg Balance Scale (BBS)	3 (40)	+ (11%)	Moderate
Mini Balance Evaluation Test (Mini BESTest)	1 (6)	+ (5%)	Very low
CAREN Specific—Boat Steering Task Score	1 (31)	+ *	Low
Tinetti’s Balance Test	1 (1)	+ (100%)	Very low
Single Leg Stance Test	1 (1)	+ (214%)	Very low
**Gait**			
Functional Gait Assessment (FGA)	5 (65)	+ (26%)	Moderate
10 Metre Walk Test (10MWT)	1 (10)	+ (9%)	Very low
CAREN Specific—Self Selected Walking Speed	1 (31)	+ *	Low
Step Variability	1 (1)	+ (9%)	Very low
**Functional**			
Timed Up and Go (TUG)	1 (9)	+ (4%)	Low
Functional Reach Test	1 (15)	+ (18%)	Low
Tinetti’s Mobility Test	1 (1)	+ (33%)	Very low
Functional Independence Measure (FIM)	1 (1)	+ (52%)	Very low
Visual Vertigo Analogue Scale	1 (15)	+ *	Very low
Eight Foot Up and Go Test	1 (15)	+ (13%)	Very low

*Studies did not report a quantifiable value, therefore the average % change could not be calculated

## Discussion

Through the evaluation of scientific literature, this scoping review found that VR is an effective therapeutic tool for vestibular and balance impairments post-concussion across all investigated categories—balance, gait, and functional. However, it remains unclear if VR is more favourable than traditional vestibular rehabilitation. A recent study with level two evidence by Sessoms and colleagues [4], compared VR vestibular rehabilitation with traditional vestibular rehabilitation. Of the four outcome measures used by Sessoms (ABC, DHI, SOT, FGA), only one reported a greater improvement with VR compared to traditional rehabilitation (SOT). Of these outcome measures, ABC and DHI are subjective measures, and the FGA is a more functional test. The SOT, however, is a highly validated measure using computerised posturography (CDP), which is sensitive to postural sway (i.e., via quantification of displacement of centre of gravity), making it the gold standard outcome measure for instrumented balance assessment [4, 52–54]. A study conducted by Meldrum [24], also deemed traditional vestibular rehabilitation to be no more beneficial for 100% of outcome measures reported, although the VR group reported more enjoyment, less tiredness and less difficulty with balance exercises. Likewise, Lei, Sunzi, and Dai, [25] support the notion that VR vestibular rehabilitation cannot achieve the same effect as traditional vestibular rehabilitation, but at least can be used as an alternative therapy. Despite these references, the findings in this scoping reviews are not undervalued, nor does it deflect from the quality of these studies. Instead, this serves as information to clinicians, showing that both traditional and VR vestibular therapy can provide patients with benefits depending on the availability, yet more definitive research is needed to ascertain the benefits of both therapy types.

There are multiple rationales to explain the positive findings of VR technology in the field of rehabilitation. Due to the range of VR modalities and capabilities, interventions can be individualised to enhance the experience dependent on presenting signs and symptoms by offering a real time multidimensional and multisensory environment [54]. Clinicians can stringently control and modify a patient’s perceived environment as well as manipulate the visual, somatosensory, and vestibular information to target specific patient deficits, with the ability to regress and progress training in real time [54]. In order to enhance neuroplasticity, therapeutic delivery should be patient-specific and allow for dynamic feedback delivered continuously during treatment that can be augmented to replicate real-life environments [55]. Cheung [56] supports this concept by stating that VR designs allow for in-depth analysis of the patient’s activity and ability, where specified real-time feedback can be provided to promote the desired neuroplastic changes. Furthermore, VR offers more abundant augmented feedback, greater opportunities for consistent task repetition, and optimal control over different practice challenge levels [57]. The consistent task repetition offered by VR therapies results in adaptations remaining long after a patient’s exposure to the virtual environment, particularly in vestibular therapy for post-concussion patients. [58]

### Balance

The findings of this scoping review support improved balance outcomes with the use of VR as a rehabilitation tool for patients with vestibular impairments post-concussion. Of the four outcome measures identified with moderate quality of evidence, three of these outcomes were categorised under balance – two subjective measures (ABC and DHI) and one objective measure (BBS). Despite this review having no good quality of evidence (modified GRADE), the findings suggest that of the current literature identified, VR is an effective rehabilitation tool for improving balance outcomes post-concussion [59–62].

Multiple studies have investigated the use of VR therapy in treating balance impairments across a wide range of disorders. A study conducted by Phu and colleagues [59], assessed balance in community-dwelling older adults at high falls risk using a fully immersive VR system. Their results found VR to be effective in improving static and dynamic balance and reducing fear of falling and fall rates over a 9-month period [59]. Furthermore, another study examined the use of VR to treat balance impairment in patients with Parkinson’s Disease (PD) [25]. The study used the BBS as an outcome measure and found that rehabilitation training based on VR technology is more effective than conventional training in improving PD patients’ balance function [1, 25].A third study is also in favour of VR treatment for balance impairments, suggesting that VR combined with conventional therapy compared to conventional therapy alone is effective in improving balance in individuals post-stroke, again using the BBS as an outcome measure [60]. All three studies support the findings in this review, that VR is a useful treatment option for treating balance impairments post-concussion.

Balance requires integration of multiple information sources (visual, vestibular, and somatosensory), adapting and reacting to both the body and the surrounding environments [61]. These behaviours are influenced by both intention-based and stimulus-based actions (feedforward and feedback) indicative of ascending as well as descending control processes [61]. Furthermore, concussion impairs central integration of balance information and reduces the ability to multi-task. Additionally, the cognitive impacts associated with concussion cause issues adapting and reaction to different environments, specifically affecting stimulus-based actions [62]. VR has been shown to positively effect balance impairments post-concussion, providing opportunities to manipulate visual information relating to oneself and the environmental characteristics [63, 64]. This is further demonstrated by the findings in this review.

The primary rationale for using VR for vestibular and balance rehabilitation is that realistic visual environments may enhance adaptation by causing retinal slip, which a recent study by Mao et al*.* [64] suggest is the key to adjusting the vestibular system. The retinal slip is the movement of a visual image across the retina, a powerful signal that can be induced by horizontal or vertical head movements while maintaining visual fixation on a target. Retinal slip can also be induced by position error signals, imagined motion of the target, and strobe lighting, all of which are possible utilising VR technology [67].

Studies show that concussion causes dysfunction of the vestibulo-ocular reflex (VOR) [64–66]. The VOR can be adapted in VR simulations with an increase in VOR gain, and VR can be used to increase the rate of adaptation by specifically adapting scenes to a person’s capability, thereby facilitating their recovery [64]. Furthermore, it has been noted that VR scenes promote rehabilitation more effectively than optokinetic-based therapies, since VR offers the ability to finely control the virtual scene [64]. Overall, the use of VR is still a relatively new concept, and though the relationship between VR and balance rehabilitation appears promising, more research is required to better understand it.

### Gait

Three separate systematic reviews [25, 68, 69] have proven the effectiveness of VR in improving gait for patients with Parkinson's disease and post-stroke patients.

The findings from this review suggest similar outcomes for patients experiencing vestibular and balance impairments post-concussion when using VR compared to conventional rehabilitation methods.VR is a useful tool in the therapy of gait training, given VR technology can change locomotion and feedback features such as speed, trajectory, and obstacle circumvention behaviours [63]. Not only is VR useful for gait outcomes in patients with vestibular and balance impairments, three separate systematic reviews [25, 68, 69] have proven the effectiveness of VR in improving gait for patients with Parkinson’s disease and post-stroke patients. Gait is reliant on concurrently integrating multiple information sources (visual, vestibular, and somatosensory), and motor commands, to adapt to contextual demands, which VR can provide and manipulate [70].

Furthermore, de Amorim and colleagues [71] highlight that VR offers opportunities for rehabilitation of weight transfer between limbs, unipedal support, triple flexion, and load acceptance during initial support, all of which are affected post-concussion. [72] However, VR is unable to provide clinicians and patients with other important aspects of gait, such as dissociation of waists, impulsion, and continuous anterior displacement of the centre of mass [71].Therefore, more research is needed to investigate the effectiveness of VR modalities to improve gait abnormalities seen in those with a concussion. [71]

Level of immersion (non-, semi- or fully immersive) can influence the effectiveness of rehabilitation for improving patients’ gait function. Recent literature [73] suggests that more immersive VR systems may bring additional benefits compared to training with less immersive VR systems due to the ability to generate a stronger feeling of ‘being physically present’. However, our findings show otherwise. Of the gait outcome measures, only one measure was used within multiple studies [4, 45, 47, 48, 51], the Functional Gait Assessment. Within these studies, the greatest improvement in FGA score was seen in the non-immersive and semi-immersive studies [45, 47]. A postulation for why, may be the restriction placed on a patients’ environment when in an immersive VR headset, making it difficult to perform gait and functional activities, compared to balance activities performed on the spot [74]. Furthermore, perhaps the cognitive load is too high with full immersion, or the feeling of ‘being physically present’ may cause symptoms of visual motion sensitivity and headaches making treatment less tolerable [73]. This highlights the need for further research to solidify which level of VR immersion might best improve gait ability post-concussion. Nevertheless, VR has the opportunity to provide cognitive challenges while walking through a virtual environment, allowing better integration of all senses within a safe environment, and perhaps central reweighting of balance information, which can be a component necessary for motion and visual motion sensitivity [47]. Furthermore, it has the potential to speed up the process of improving spatial disorientation back to a level of high functional performance [47].

This scoping review shows some moderate evidence supporting improvement in the functional gait assessment (FGA) of > 25%. However, this percentage could be higher if VR were able to provide the opportunity to cover all aspects of gait, including abnormal acute single-task simple gait, poor subacute balance control during dual-task gait, and subacute gait abnormalities during specific complex gait tasks [71, 72]. This could be possible if gait rehabilitation combined VR with other technologies such as a traditional treadmill or omnidirectional treadmill [75]. A study [76] conducted on the healthy population investigated the effect of VR during both fixed speed and self-paced treadmill walking. At fixed speed walking, the use of VR on a treadmill led to slightly improved gait pattern, while at self-paced walking, patients altered their gait technique to maximise stability. However, the effects found were too small to be clinically relevant. A further study [77] investigated the effects of treadmill training with VR on gait in children with cerebral palsy. Results showed that gait velocity and walking endurance improved to greater extents in the VR treadmill training group compared to treadmill training group. These findings promote the combination of VR with other training methods to maximise gait rehabilitation. The evidence is currently limited, and non-specific to post-concussion patients, however, based on the low quality of evidence found in this review, further research into best practice for VR for gait outcomes is necessary.

### Functional

Current literature [78, 79] has demonstrated that VR is an effective tool to support and improve activities of daily living (ADLs) and functional ability of older adults in comparison to traditional rehabilitation. The findings from this scoping review suggest that these effects are also present in patients with vestibular and balance impairments post-concussion.

The findings for the functional outcomes saw improvements of > 50%, suggesting VR is useful in targeting specific day-to-day tasks. However, these findings were all very low to low quality, highlighting the urge for further research to ascertain effectiveness of VR for functional outcome measures. The low quality could be due to the fact that functional outcome measures can be harder to standardise. Despite the low-quality evidence, these findings still demonstrate improvement in functional measures using VR as a rehabilitation tool. This is possibly due to VR technologies being able to address functional rehabilitation goals given it can simulate real world environments, allowing for specific impairments to be trained within a functional context [20, 46]. Within a virtual environment, practiced tasks can replicate real-world conditions and the choice of scenes can be based on functional needs of the patient, thus applicability and transferability to daily living is more likely [20]. This is an advantage to clinicians, given the opportunity for rehabilitation flexibility in adapting the exercises specifically to patients’ needs, while the patient remains in a safe environment. Of the limited research available discussing the functional benefits of VR, the overarching argument is that VR-based interventions relying on functional tasks offers the potential for more effective rehabilitation given the training is conducted in more naturalistic settings than traditional interventions [79].

### Gaps in the literature

After reviewing the current literature, the authors have identified gaps and therefore the potential for future research. Despite having a wide array of search terms, the authors only managed to find ten suitable studies, all within the past nine years. It was noticeable that current research tends to focus on the assessment role of VR for vestibular and balance impairments post-concussion [26–30], despite there being opportunities to extend the same principles for the training of skills required for successful improvements for patients following a concussion [80]. Therefore, to further make conclusions about the use of VR in rehabilitation, and provide clinicians with robust evidence on its benefits, more prospective research is required.

The current research lacks detail on the most appropriate way of applying VR intervention and intensity suitability in a program according to the severity of concussion experienced by patients [64]. Future research would benefit from exploring the mechanism of the integrated central and peripheral nervous system, visual, vestibular and proprioceptive sensation, and more detailed clinical techniques for the use of VR in rehabilitation [64]. Establishing a quantitative standard of VR intervention is highly warranted and will improve the operational feasibility of VR. Likewise, clinicians would benefit from research surrounding the feasibility of VR in a home-based setting and if a home-based intervention has the same validity and clinical relevance as in clinical settings.

## Strengths and limitations

Within this scoping review were several strengths allowing the authors to accurately capture the volume of scientific literature and types of evidence surrounding VR for post-concussion patients with vestibular symptoms. The eligibility criteria were carefully established, allowing for identification and analysis of a wide range of current literature, to increase the external validity of the results. All included studies in this scoping review were conducted recently (in the last ten years). Although VR in vestibular and balance rehabilitation is still a relatively new concept, comparing recent studies allows clinicians to utilise these methods as they are still relevant within the field. An additional strength of this review was the modified GRADE system which is tailored to suggesting quality of evidence in a way that is easy for clinicians and researchers to grasp. A modified system was chosen as the GRADE handbook recommends that authors comment only on the strength of articles, not overall quality of evidence, leaving that to panels of objective judges to consider. However, in order to succinctly demonstrate areas where more research is necessary for this scoping review, the authors saw that this tool was necessary and appropriate.

Whilst these findings are of interest, they are not without limitations. The major confounder for this review lies within the current literature and the reporting of results, specifically with the effect size, statistical significance, and the inconsistent use of outcome measures. Of the ten studies, only three [4, 50, 51] reported significance (p values)and none of the studies reported an effect size. These factors limit determining observed effect and if the effect arose due to chance, ultimately restricting the internal and external validity. Ultimately, the small number of studies and heterogeneity of data made it too challenging to complete a true systematic review or meta-analysis.

Additionally, within the current literature, reported outcome measures varied significantly revealing nineteen different measures over ten studies, where only six were reported in more than one study. This heterogeneity between studies created uncertainty when summarising the applicability and clinical interpretation of results. Due to most studies using various outcomes, the authors were able to comment on the trends of treatment but were not able to quantify the effectiveness of VR in treating vestibular and balance impairments post-concussion. Further, due to the limited research available, no consensus in which type of VR (immersive, semi-immersive or non-immersive) is most effective could be achieved.

For these reasons authors reported on % change per hour of exposure, albeit identifying in the results that the duration of intervention lasted from fifteen to sixty minutes.

This unit of measurement was utilised to highlight a standardised improvement effect across outcomes, however the authors understand that clinically this would not be beneficial or attainable, as an hour of therapy is commonly not achievable for all individuals. Patients experiencing vestibular impairments may not be able to sustain intervention for a consecutive hour [49]. Session lengths are variable and very symptomatically dependent. The indicative factors affecting therapy session length may include sleep, emotions, and autonomic changes. Comparably, in Rabago and Wilken’s study [49], the length of intervention session was sixty minutes in duration, but further in the report they also stated that most treatment bouts were restricted to a 10 min duration. This statement further adds to the limitation that not all studies are clear on the true duration of intervention exposure.

## Conclusion

This scoping review shows that VR is an effective tool for the rehabilitation of vestibular and balance impairments post-concussion. It provides detailed information about the current literature available and may serve as a reference for clinicians or future directions. VR is effective in improving balance, gait, and functional outcome in patients post-concussion. Of the ten studies included, all 19 outcome measures found positive effects when using VR for rehabilitation. Most outcome measures were categories into balance (nine of the 19), with four in gait and six in functional. Despite the positive effects of VR, the quality of evidence across all found literature was of low grade, highlighting the need for better quality evidence for the use of VR in the rehabilitation of patients post-concussion. Future research may be justified to establish a quantitative standard of VR intervention, and further investigate the relative effects of VR in vestibular and balance rehabilitation post-concussion.

## Supplementary Information

**Additional file 1**. Search terms used for all databases.

**Additional file 2**. Data charting from the ten selected studies.

## Data Availability

The datasets used and analysed during the current study are available from the corresponding author on reasonable request.

## Abbreviations

ABC Scale: Activities-Specific Balance Confidence Scale
ADL: Activities of daily living
BBS: Berg Balance Scale
CAREN: Computer Assisted Rehabilitation Environment
CAS: Critical Appraisal Scores
DHI: Dizziness Handicap Inventory
FGA: Functional Gait Assessment
FSST: Four-Square Step Test
GRADE: Grading of Recommendations, Assessment, Development and Evaluation
JBI: Joanna Briggs Institute
mTBI: Mild traumatic brain injury
OSF: Open science framework
PRISMA: Preferred Reporting Items for Systematic Reviews and Meta-Analyses
PRISMA-P: Preferred Reporting Items for Systematic Reviews and Meta-Analyses protocols
PRISMA-ScR: Preferred Reporting Items for Systematic Reviews and Meta-Analyses for Scoping Reviews
RCT: Randomised control trials
SOT: Sensory Organisation Test
SRA: Systematic review accelerator
VOR: Vestibulo-ocular reflex
VR: Virtual reality
