# Supplementary material for: Virtual reality-based interventions for the rehabilitation of vestibular and balance impairments post-concussion: a scoping review
Source: J Neuroeng Rehabil. 2023 Mar 3;20:31. doi: 10.1186/s12984-023-01145-4 (PMC9985280; doi:10.1186/s12984-023-01145-4)
Supplement: Supplementary file 1 — Additional file 1. Search terms used for all databases. [file 12984_2023_1145_MOESM1_ESM.docx]

**Additional file 1**

**Search terms used for all databases.**

| Database | Search Terms |
| --- | --- |
| PubMed | ("virtual reality"[tiab] OR "immersive technology"[tiab] OR "augmented reality"[tiab] OR "computer simulation"[tiab] OR simulation[tiab] OR "Virtual Reality"[Mesh]) AND (concussion[tiab] OR "traumatic brain injury"[tiab] OR (head[tiab] AND injury[tiab] OR trauma[tiab]) OR "Brain Concussion"[Mesh] OR post-concussion[tiab] OR TBI[tiab]) AND (vestibular[tiab] AND Disorders[tiab] OR therap*[tiab] OR rehabilita*[tiab] OR management[tiab]) |
| Embase | ('virtual reality':ti,ab OR 'immersive technology':ti,ab OR 'augmented reality':ti,ab OR 'computer simulation':ti,ab OR simulation:ti,ab OR 'virtual reality'/exp OR 'virtual reality') AND (concussion:ti,ab OR 'traumatic brain injury':ti,ab OR (head:ti,ab AND injury:ti,ab) OR trauma:ti,ab OR 'brain concussion'/exp OR 'brain concussion' OR 'post concussion':ti,ab OR tbi:ti,ab) AND (vestibular:ti,ab AND disorders:ti,ab OR therap*:ti,ab OR rehabilita*:ti,ab OR management:ti,ab) |
| Scopus | ((TITLE-ABS("virtual reality") OR TITLE-ABS("immersive technology") OR TITLE-ABS("augmented reality") OR TITLE-ABS("computer simulation") OR TITLE-ABS(simulation) OR INDEXTERMS("Virtual Reality")) AND (TITLE-ABS(concussion) OR TITLE-ABS("traumatic brain injury") OR (TITLE-ABS(head) AND TITLE-ABS(injury) OR TITLE-ABS(trauma)) OR INDEXTERMS("Brain Concussion") OR TITLE-ABS(post-concussion) OR TITLE-ABS(TBI))) AND (TITLE-ABS(vestibular) AND TITLE-ABS(Disorders) OR TITLE-ABS(therap*) OR TITLE-ABS(rehabilita*) OR TITLE-ABS(management)) |
| CINAHL | ((TI "virtual reality" OR AB "virtual reality") OR (TI "immersive technology" OR AB "immersive technology") OR (TI "augmented reality" OR AB "augmented reality") OR (TI "computer simulation" OR AB "computer simulation") OR (TI simulation OR AB simulation)OR (MH "Virtual Reality"+)) AND ((TI concussion OR AB concussion) OR (TI "traumatic brain injury" OR AB "traumatic brain injury") OR ((TI head OR AB head) AND (TI injury OR AB injury) OR (TI trauma OR AB trauma)) OR (MH "Brain Concussion"+) OR (TI post-concussion OR AB post-concussion) OR (TI TBI OR AB TBI))) AND ((TI vestibular OR AB vestibular) AND (TI Disorders OR AB Disorders) OR (TI therap* OR AB therap*) OR (TI rehabilita* OR AB rehabilita*) OR (TI management OR AB management)) |
| SportDiscus | (((TI "virtual reality" OR AB "virtual reality") OR (TI "immersive technology" OR AB "immersive technology") OR (TI "augmented reality" OR AB "augmented reality") OR (TI "computer simulation" OR AB "computer simulation") OR (TI "simulation" OR AB "simulation") OR DE "Virtual Reality") AND ((TI "concussion" OR AB "concussion") OR (TI "traumatic brain injury" OR AB "traumatic brain injury") OR ((TI "head" OR AB "head") AND (TI "injury" OR AB "injury") OR (TI "trauma" OR AB "trauma")) OR DE "Brain Concussion" OR (TI "post-concussion" OR AB "post-concussion") OR (TI "TBI" OR AB "TBI"))) AND ((TI "vestibular" OR AB "vestibular") AND (TI "Disorders" OR AB "Disorders") OR (TI "therap*" OR AB "therap*") OR (TI "rehabilita*" OR AB "rehabilita*") OR (TI "management" OR AB "management")) |
| Proquest | (TI,AB("virtual reality") OR TI,AB("immersive technology") OR TI,AB("augmented reality") OR TI,AB("computer simulation") OR TI,AB(simulation) OR MESH.EXACT.EXPLODE("Virtual Reality")) AND (TI,AB(concussion) OR TI,AB("traumatic brain injury") OR (TI,AB(head) AND TI,AB(injury) OR TI,AB(trauma)) OR MESH.EXACT.EXPLODE("Brain Concussion") OR TI,AB(post-concussion) OR TI,AB(TBI)) AND (TI,AB(vestibular) AND TI,AB(Disorders) OR TI,AB(therap*) OR TI,AB(rehabilita*) OR TI,AB(management)) |
