# Supplementary material for: Virtual reality-based interventions for the rehabilitation of vestibular and balance impairments post-concussion: a scoping review
Source: J Neuroeng Rehabil. 2023 Mar 3;20:31. doi: 10.1186/s12984-023-01145-4 (PMC9985280; doi:10.1186/s12984-023-01145-4)
Supplement: Supplementary file 2 — Additional file 2. Data charting from the ten selected studies. [file 12984_2023_1145_MOESM2_ESM.docx]

**Additional file 2**

**Data charting of the included studies.**

|  |  |  |  | **Results** | | | | | | |
| --- | --- | --- | --- | --- | --- | --- | --- | --- | --- | --- |
| **Author & Year** | **Study Design** | **Duration of Intervention** | **Outcome Measure** | **Pre** | **Post** | **Change** | **Change over time (hrs)** | **Statistical Change** | **Relative % Change** | **% Change/ Time (hrs of intervention)** |
| Cano Porras et al.  2019 ^36^ | Retrospective Cohort Study | 12 sessions 30–45 mins each 9h total | 10 Metre Walk Test -ST | 0.68 ± 0.14 | 0.74 ± 0.15 | -0.06 | 0 | Improved | 9% | 1.00% |
|  |  |  | 10 Metre Walk Test -DT | 0.76 ± 0.14 | 0.75 ± 0.10 | 0.01 | 0.00 | Improved | 1% | 0.11% |
|  |  |  | Timed Up and Go -ST | 27.62 ± 11.70 | 26.63 ± 11.05 | 0.99 | 0.11 | Improved | 4% | 0.44% |
|  |  |  | Timed Up and Go -DT | 18.52 ± 3.21 | 17.16 ± 1.97 | 1.36 | 0.15 | Improved | 7% | 0.08% |
|  |  |  | Four Square Step Test | 19.13 ± 6.32 | 15.36 ± 4.87 | 3.77 | 0.42 | Improved | 20% | 2.22% |
|  |  |  | Berg Balance Scale | 26 ± 5.94 | 29.00 ± 9.55 | -3.00 | -0.33 | Improved | 12% | 1.33% |
|  |  |  | Mini Balance Evaluation Test | 14.83 ± 2.21 | 15.5 ± 2.83 | -0.67 | -0.07 | Improved | 5% | 0.56% |
| Cuthbert et al.  2012 ^37^ | A Pilot Parallel Randomised Control Trial | 6 weeks 24 sessions Four times per week 15 min each 6h total | Berg Balance Scale | 47.21  (42.60, 51.82) | 52.57  (47.87, 57.26) | 5.36 | 3.57 | No change | 11% | 1.83% |
|  |  |  | Functional Gait Assessment | 16.36  (12.22, 20.50) | 22.85  (18.66, 27.05) | 6.49 | 4.33 | No change | 40% | 6.67% |
| De Luca et al.  2019 ^38^ | Case Report | 2 programs (Conventional Cognitive Training & CAREN) 4 weeks / 20 sessions Five times per week 45 mins each 15h total 3 week break between programs | Tinetti’s Mobility Test | 9 | 12 | 3 | 0.2 | Improved | 33% | 2.22% |
|  |  |  | Tinetti's Balance Test | 8 | 16 | 8 | 0.53 | Improved | 100% | 6.67% |
|  |  |  | Functional Independence Measure - Motor Abilities | 60 | 91 | 31 | 2.07 | Improved | 52% | 3.44% |
|  |  |  |  |  |  |  |  |  |  |  |
| Gottshall & Sessoms  2015 ^39^ | Case Report | 3 weeks 3 sessions 45 mins each 2.25h total | Dizziness Handicap Inventory | 12 | 0 | 12 (%) | 5.33 | Improved | 100% | 44.44% |
|  |  |  | Activities Specific Balance Confidence Scale | 78 | 85 | 7 (%) | 3.11 | Improved | 9% | 3.99% |
|  |  |  | Functional Gait Assessment | 23 | 30 | 7 | 3.11 | Improved | 30% | 13.53% |
|  |  |  | Sensory Organisation Test | 71 | 84 | 13 (%) | 5.78 | Improved | 18% | 8.14% |
| Gottshall, Sessoms & Bartlett  2012 ^40^ | Quasi-Experimental Design | 6 weeks 15 sessions No session length specified | Sensory Organisation Test | 66.75 | 68.25 | 1.5 | - | Improved | 2% | - |
|  |  |  | Functional Gait Assessment | 25 | 30 | 5 | - | Improved | 20% | - |
|  |  |  | Dizziness Handicap Inventory | 42.5 | 16.5 | 26 | - | Improved | 61% | - |
|  |  |  | Activities Specific Balance Confidence Scale | 70.75 | 85.25 | 14.5 | - | Improved | 20% | - |
| Lubetzky et al.  2020 ^12^ | Quasi-Experimental Design | 3-8 sessions Average of 6 sessions 30-45 mins each 4.5h total | Visual Vertigo Analogue Scale | - | - | 7.5 | 1.67 | Improved | - | - |
|  |  |  | Dizziness Handicap Inventory | - | - | 10 | 2.22 | Improved | - | - |
|  |  |  | Activities Specific Balance Confidence Scale | - | - | 15 | 3.33 | Improved | - | - |
|  |  |  | Eight Foot Up and Go | 6.85 | 5.93 | 0.92 | 0.20 | Improved | 13% | 2.985% |
|  |  |  | Four Square Step Test | 11 | 1.67 | 9.33 | 2.07 | Improved | 85% | 18.848% |
| Rábago & Wilken  2011 ^41^ | Case Report | 6 weeks 6 sessions 60 mins each 6h total | Single Leg Stance Test (EC, L) | 3.9 | 18.1 | 14.2 | 2.367 | Improved | 364% | 60.68% |
|  |  |  | Single Leg Stance Test (EC, F) | 2.8 | 5.5 | 2.7 | 0.450 | Improved | 96% | 16.07% |
|  |  |  | Single Leg Stance Test (EO, L, NF) | 13.6 | 28.7 | 15.1 | 2.517 | Improved | 111% | 18.50% |
|  |  |  | Single Leg Stance Test (EO, L, OF) | 14.8 | 26.8 | 12 | 2.000 | Improved | 81% | 13.51% |
|  |  |  | Single Leg Stance Test (EO, F, OF) | 4.4 | 22.8 | 18.4 | 3.067 | Improved | 418% | 69.70% |
|  |  |  | Step Variability (Platform Perturbations) | 8.5 | 7.7 | 0.8 | 0.133 | Improved | 9% | 1.57% |
|  |  |  | Step Variability (Visual Perturbations) | 10 | 4.1 | 5.9 | 0.983 | Improved | 59% | 9.83% |
| Sessoms et al.  2015 ^42^ | Randomised Control Trial | 6 weeks 12 sessions Twice per week 30 mins 6h total | CAREN Specific Outcome Measures - Self Selected Walking Speed | - | - | - | - | Improved | - | - |
|  |  |  | CAREN Specific Outcome Measures - Boat Steering Task Score | - | - | - | - | Improved | - | - |
| Sessoms et al.  2021 ^4^ | A Pilot Parallel Randomised Control Trial | 6 weeks 12 sessions Twice per week 30 mins 6h total | Activities Specific Balance Confidence Scale | 75.5 | 90.05 | -14.55 | -2.43 | Improved | 19% | 3.17% |
|  |  |  | Dizziness Handicap Inventory | 36.42 | 18.92 | 17.50 | 2.92 | Improved | 48% | 8% |
|  |  |  | Sensory Organisation Test | 64.08 | 74.25 | 10.17 | 1.70 | Improved | 16% | 2.67% |
|  |  |  | Functional Gait Assessment | 25.25 | 29.33 | 4.08 | 0.68 | Improved | 16% | 2.67% |
| Ustinova et al.  2014 ^43^ | Quasi-Experimental Design | 5-6 weeks 30.75h total | Berg Balance Scale | 45.6 | 50.2 | 4.6 | 0.15 | Improved | 10% | 0.33% |
|  |  |  | Functional Gait Assessment | 20.3 | 24.9 | 4.6 | 0.15 | Improved | 23% | 0.75% |
|  |  |  | Functional Reach Test | 12.5 | 14.8 | 2.3 | 0.07 | Improved | 18% | 0.59% |

- , Data not recorded

Min, minutes; hrs, hours; m/s, meters per second; s, seconds; Change, change in pre verses post intervention scores

10MWT-ST, 10 Meter Walk Test Single Task; 10MWT-DT, 10 Meter Walk Test Dual Task; TUG-ST, Timed up and Go Single Task; TUG-DT, Timed up and Go Dual Task; Mini-BESTest, Mini Balance Evaluation Systems Test; CAREN, Computer Assisted Rehabilitation Environment
